# “*I know you didn’t want to stay*”: Emergency Department Conversations about Disposition for People Living with Dementia

**DOI:** 10.1002/alz70858_099393

**Published:** 2025-12-24

**Authors:** Justine Seidenfeld, Matthew Tucker, Melissa Harris‐Gersten, Gemmae M Fix, Nina R Sperber, Susan Nicki Hastings

**Affiliations:** ^1^ Durham VA Healthcare System, Durham, NC, USA; ^2^ VA Bedford Healthcare System, Bedford, MA, USA

## Abstract

**Background:**

When people living with dementia present to the emergency department (ED), the disposition choice‐ to admit them to the hospital or discharge them home‐ can be difficult for providers. In cases that are not straightforward, input from people living with dementia and their care partners may improve the quality of disposition decision‐making. However, little is known about current real‐world practices in ED disposition conversations.

**Method:**

This ethnographic study used direct observations of ED encounters with people living with dementia, their care partners, and ED providers at a Veterans Affairs Medical Center. Follow up interviews were conducted with patients and care partners who were observed. Interview guides were informed by the Ottawa Decision Support Framework, and prompts informed by observations. Data were analyzed using the constant comparative method.

**Result:**

Data was collected over 45 days, with 20 ED encounters,18 follow‐up interviews, and baseline survey data. For the 20 participants living with dementia, all were male, mean age was 79.4, and 50% were Black or African American. Major themes included 1) significant variation in the depth of disposition conversations, 2) patient and care partner participation varied with disposition outcome, and 3) satisfaction with disposition decision‐making was mostly driven by alignment of preferences.

**Conclusion:**

Our study suggests that there are no consistent formats of ED disposition conversations for people living with dementia. Improving quality may be most needed when disposition preferences are misaligned. Given variation in preferences, they should be identified early in the ED disposition conversation.

